# Induction of Apoptosis, Autophagy and Ferroptosis by *Thymus vulgaris* and *Arctium lappa* Extract in Leukemia and Multiple Myeloma Cell Lines

**DOI:** 10.3390/molecules25215016

**Published:** 2020-10-29

**Authors:** Aveen N. Adham, Mohamed Elamir F. Hegazy, Alaadin M. Naqishbandi, Thomas Efferth

**Affiliations:** 1 Department of Pharmacognosy, College of Pharmacy, Hawler Medical University, Erbil 44001, Kurdistan Region, Iraq; aveen.adham@hmu.edu.krd; 2 Department of Pharmaceutical Biology, Institute of Pharmaceutical and Biomedical Sciences, Johannes Gutenberg University, Staudinger Weg 5, 55128 Mainz, Germany; mohegazy@uni-mainz.de; 3 Chemistry of Medicinal Plants Department, National Research Centre, 33 El-Bohouth St., Dokki, Giza 12622, Egypt

**Keywords:** apoptosis, asteraceae, autophagy, cell death, lamiaceae, ferroptosis, multiple myeloma, phytotherapy

## Abstract

*Thymus vulgaris* and *Arctium lappa* have been used as a folk remedy in the Iraqi Kurdistan region to deal with different health problems. The aim of the current study is to investigate the cytotoxicity of *T. vulgaris* and *A. lappa* in leukemia and multiple myeloma (MM) cell lines and determine the mode of cell death triggered by the most potent cytotoxic fractions of both plants in MM. Resazurin assay was used to evaluate cytotoxic and ferroptosis activity, apoptosis, and modulation in the cell cycle phase were investigated via Annexin V-FITC/PI dual stain and cell-cycle arrest assays. Furthermore, we used western blotting assay for the determination of autophagy cell death. *n*-Hexane, chloroform, ethyl acetate, and butanol fractions of *T. vulgaris* and *A. lappa* exhibited cytotoxicity in CCRF-CEM and CEM/ADR 5000 cell lines at concentration range 0.001–100 μg/mL with potential activity revealed by chloroform and ethyl acetate fractions. NCI-H929 displayed pronounced sensitivity towards *T. vulgaris* (TCF) and *A. lappa* (ACF) chloroform fractions with IC_50_ values of 6.49 ± 1.48 and 21.9 ± 0.69 μg/mL, respectively. TCF induced apoptosis in NCI-H929 cells with a higher ratio (71%), compared to ACF (50%) at 4 × IC_50_. ACF demonstrated more potent autophagy activity than TCF. TCF and ACF induced cell cycle arrest and ferroptosis. Apigenin and nobiletin were identified in TCF, while nobiletin, ursolic acid, and lupeol were the main compounds identified in ACF. *T. vulgaris* and *A. lappa* could be considered as potential herbal drug candidates, which arrest cancer cell proliferation by induction of apoptosis, autophagic, and ferroptosis.

## 1. Introduction

Hematologic malignancies are sorts of cancer that originate in the blood-forming tissue such as the bone marrow or the lymph system, and include leukemia, lymphoma, and multiple myeloma (MM) [1]. MM is one of the most common types of hematological cancer and globally accounts for about 10% of all hematologic malignancies. MM is characterized by the accumulation of atypical plasma cells in the bone marrow associated with abnormal production of monoclonal immunoglobulins, which triggers renal complications, hypercalcemia, severe bone pain and destruction, and anemia. The disease is more prevalent among males than females and more in elderly persons [2]. Leukemia is another life-threatening hematological malignancy, characterized by abnormal elevation number of leucocytes in the blood and bone marrow, resulting from a combination of environmental and genetic factors [3]. Leukemia occurs in adults over the age of 55 years but is the most predominant cancer in children younger than 15 years of age. Among the most common types of leukemia are acute lymphoblastic leukemia and acute myeloid leukemia with a five-year survival rate of 68.2 and 26.9%, respectively [4]. Medicinal plants have drawn great attention as a source for novel oncological therapeutics due to their bioactive chemical ingredients with potential effectiveness and minimal toxicity profiles [5].

*Thymus vulgaris* is a perennial pleasant-smelling plant of the mint family Lamiaceae and commonly known as thyme. The Kurdish name of the plant is Jatre. The plant grows in coarse, rough soils and sunny climates. It is native to Asia, Europe, America, and Africa [6], and since ancient times has been used as a condiment, perfume, and incense [7]. The plant is known for its essential oil content such as (thymol, carvacrol, β-myrcene, γ-terpinene, linalool, terpinene-4-ol, p-cymene), flavonoids (apigenin, thymonin, luteolin-7-*O*-glucuronide, luteolin-7-*O*-rutinoside, eriodictiol-7-*O*-rutinoside, nobiletin, cirsilineol, and 8-methoxycirsilineol), quinones (arbutin), and phenolic acid (caffeic acid and rosmarinic acid) [8]. Thyme possesses various biological activities including anti-viral, anti-inflammatory, anti-oxidant, anti-cancer, insecticidal, antidiabetic, and anti-spasmodic activities [9]. *T. vulgaris* possess a hepatoprotective effect against acetaminophen-induced hepatic necrosis in mice [10]. According to numerous studies, *T. vulgaris* inhibited the viability of various tumor cell lines in a concentration-dependent manner such as breast cancer, oral cavity squamous cell carcinoma, leukemia, prostate carcinoma, cervical epithelial carcinoma, and lung carcinoma [11,12]. The human colorectal HCT116 cancer cell model was shown to prevent the rate of cell proliferation and stimulated apoptosis associated with increased caspase-3/7 activity [13].

*Arctium lappa* is a biennial edible flowering plant of the family Asteraceae and commonly known as burdock. The Kurdish name of the plant is Bnawatom. It is found in woods and forests, but mainly alongside roads, waste places, and rivers. It is cultivated in the Hawraman region, southern Kurdistan, Iraq as a medicinal plant [14]. *A. lappa* is native to Europe and Asia and rapidly spread across North America by the early European settlers [15]. The roots of *A. lappa* contain diverse bioactive secondary metabolites such as lignans (arctigenin, arctiin, and diarctigenin), polyphenols (caffeic acid, caffeic acid 4-o-glucoside, chlorogenic acid, quercitrin, quercetin, quercetin-3-*O*-glucuronide, nobiletin, p-coumaric acid, biachanin A, and tangeretin), tannin, and terpenoids (lupeol, ursolic, and oleanolic acids) [16]. These ingredients are known for their free-radical scavenging activity, anti-cancer, anti-metastatic, anti-allergic, anti-inflammatory, anti-hepatotoxic and anti-viral potency [17]. Tian, X. et al., reported the neuroprotective effects of the ethyl acetate extract of *A. lappa* roots against H_2_O_2_ induced cell damage in human neuroblastoma SH-SY5Y cells [18]. Investigation of the effects of *A. lappa* on human cancer cells showed that on the treatment of cells with different extracts, dichloromethane extracts revealed activity, especially for leukemia K562, breast MCF-7 and renal 786-0 cell lines with tumor growth inhibition at 3.62, 41.1, and 60.32 μg/mL, respectively [19].

*T. vulgaris* and *A. lappa* are among the commonly used traditional medicines in Iraq for treatment of diseases related to cancer or that may lead to cancer, such as skin diseases, blood-related diseases, inflammatory diseases, immune disorders, and infectious diseases [14,20]. There is little scientific evidence on the cytotoxic activity of *T. vulgaris* and *A. lappa* towards MM cell lines. Therefore, the goal of the present investigation was to evaluate the cytotoxicity of *T. vulgaris* and *A. lappa* extracts against various MM cell lines, to elucidate the mechanisms of cell death and to identify the bioactive compounds present in the most effective extracts.

## 2. Results

### 2.1. Cytotoxicity of T. vulgaris and A. lappa

Butanol and ethyl acetate extracts revealed the best extraction yields among the four extract types in both plants followed by *n*-hexane and chloroform extracts, (Table 1).

The results of the resazurin assay displayed that all fractions exhibited cytotoxic activity in an inhibitory concentration 50 (IC_50_) range from 2.13 ± 3.77 µg/mL (chloroform fraction (CF) in CCRF-CEM cells) to 94.35 ± 4.60 µg/mL (butanol fraction (BF) in CEM/ADR5000 cells). Among the fractions tested, chloroform and ethyl acetate revealed the highest cytotoxic activity against both cell lines (Table 2). Multidrug-resistant CEM/ADR5000 cells revealed only low degrees of cross-resistance to ethyl acetate fraction (EF), CF and BF of *T. vulgaris* and *n*-hexane fraction (HF), CF and BF of *A. lappa* (range of resistance degrees from 1.88 to 5.71) and were not cross-resistant to HF of *T. vulgaris* and EF of *A. lappa* (degrees of resistance: 1.08 and 1.18). For comparison, CEM/ADR5000 cells exhibit high level cross-resistance to its selection agent, doxorubicin, of more than 1000 and high-level cross-resistant to other natural product-derived anticancer drugs (other anthracyclines, Vinca alkaloids, taxanes, and epiodophyllotoxins) [21].

Both fractions were further investigated against 9 MM cell lines (Table 3). *Thymus* chloroform fraction (TCF) and *Arctium* chloroform fraction (ACF) demonstrated greatest growth inhibitory activity compared to *Thymus* ethyl acetate fraction (TEF) and *Arctium* ethyl acetate fraction (AEF) against all examined MM cancer cell lines, especially NCI-H929 cells for TCF (IC_50_: 6.49 ± 1.48 μg/mL) and RPMI-8226 for ACF (IC_50_: 18.26 ± 0.26 μg/mL).

**Figure 1:**
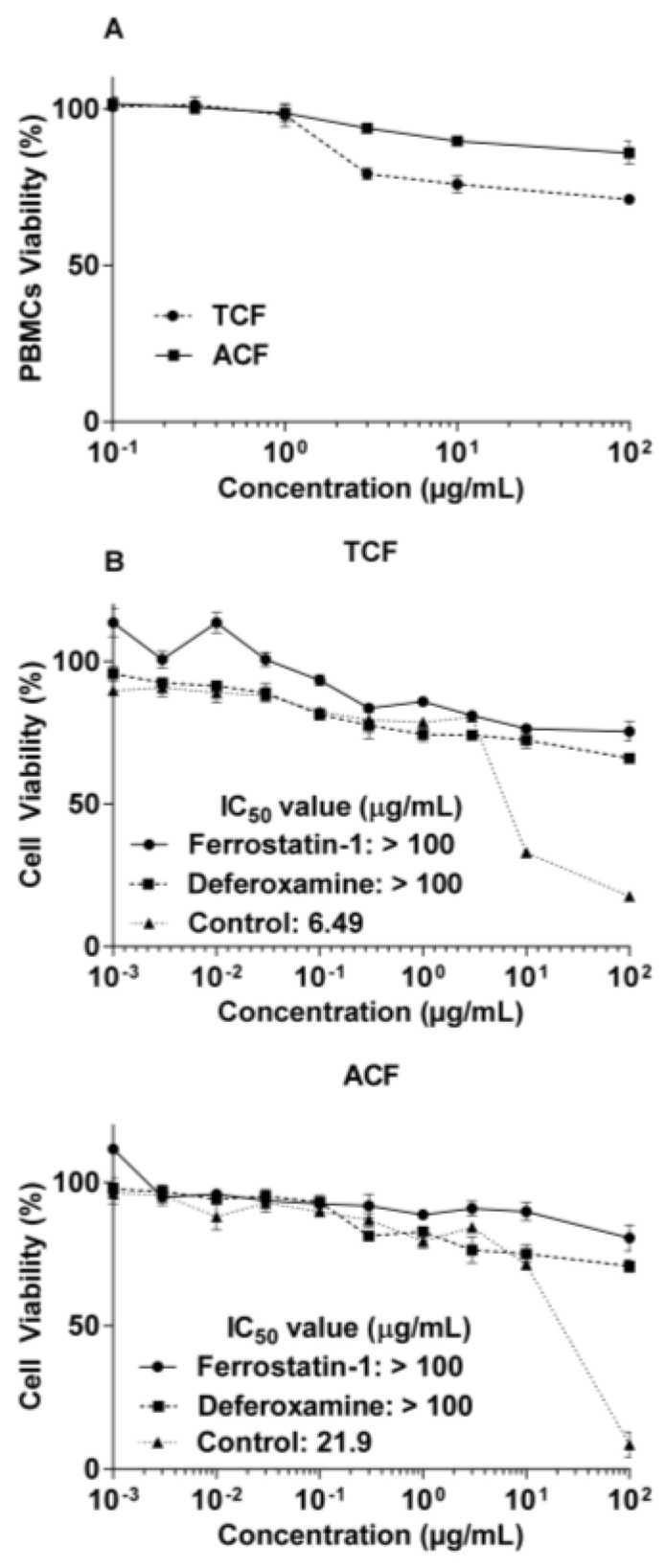
Cytotoxicity of chloroform fractions of *T. vulgaris* (TCF) and *A. lappa* (ACF) towards NCI-H929 cells and peripheral blood mononuclear cells (PBMCs) as determined by the resazurin assay. (**A**): Cytotoxicity towards normal PBMCs. (**B**): The ferroptosis inhibitors (ferrostatin-1 and deferoxamine) abrogated cytotoxicity of the extracts, indicating the role of ferroptosis cell death. Control: NCI-H929 cell without ferroptosis inhibitors.

Compared to leukemia and MM cell lines, these extracts did not display cytotoxicity against non-cancerous peripheral blood mononuclear cells (PBMCs) up to 100 µg/mL (Figure 1A). TEF showed the lowest activity in MOLP-8 (IC_50_: 41.63 ± 0.53 µg/mL) and AEF demonstrated no obvious cytotoxic effect of up to 100 μg/mL in RPMI-8226 cells after 72 h incubation.

The data in Figure 1b demonstrate the influence of ferroptosis inhibitors. Both ferrostatin-1 and deferoxamine nullified the cytotoxic activity of TCF and ACF up to the highest concentration tested (100 μg/mL).

### 2.2. Apoptosis via Intracellular ROS Generation and MMP Disruption

**Figure 2:**
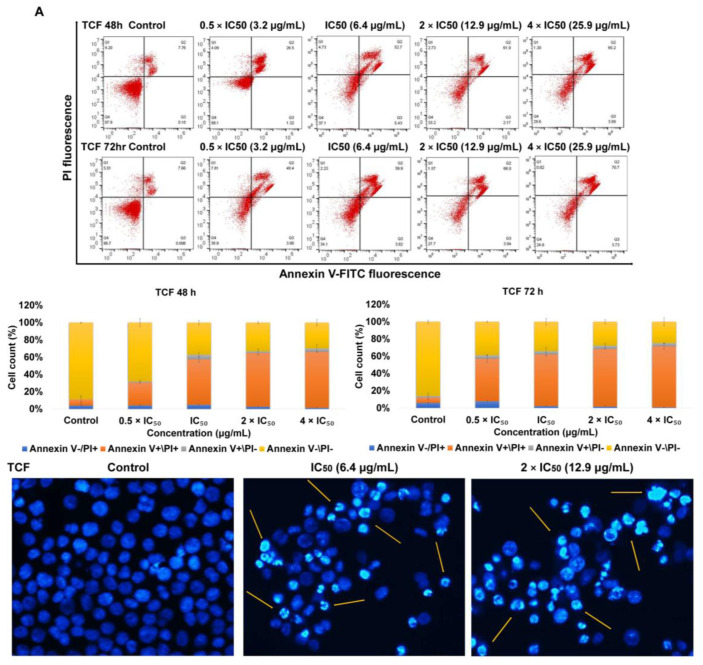

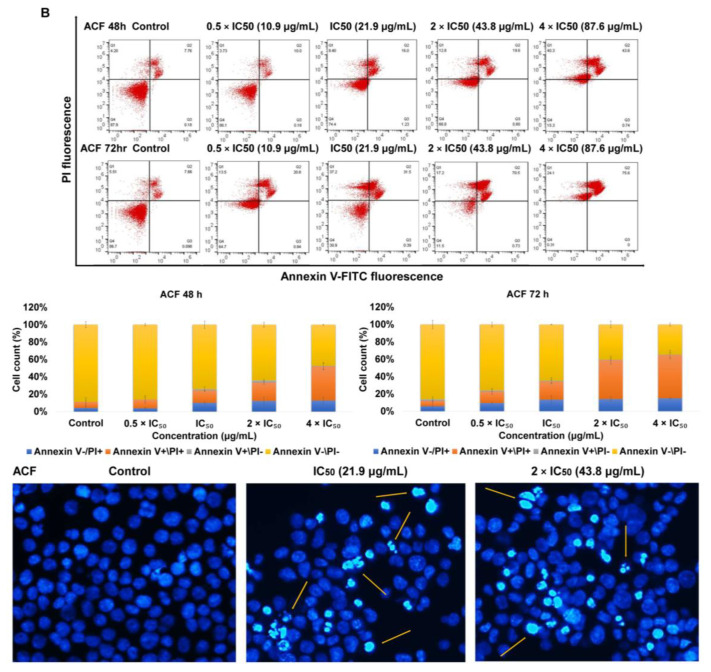
Apoptosis induction in NCI-H929 cell lines. Annexin V/PI assay of NCI-H929 cells incubated with 0.5, 1, 2, 4 × IC_50_ of chloroform fractions of (**A**)-*T. vulgaris* (TCF) and (**B**)-*A. lappa* (ACF) for 48 and 72 h. Morphology of NCI-H929 cell was noticed under fluorescence microscope by using 4′,6-diamidino-2-phenylindole (DAPI) stain. Yellow arrows referred to apoptotic features were observed in **A**-TCF and **B**-ACF treated cells vs. untreated cells. Control: NCI-H929 cell without treatment.

Annexin V-FITC/PI staining was used to determine whether apoptosis as a mode of cell death is involved in TCF- and ACF-promoted growth inhibition in NCI-H929 cells. As shown in Figure 2, the treatment of NCI-H929 cells with TCF induced late apoptosis (66% and 71% vs. 6% and 5% for untreated cells). On the other hand, exposure to ACF also induced late apoptosis (40% and 50% vs. 6% and 5% for untreated cells) and late necrosis (13% and 17% vs. 4% and 6% for untreated cells). For visualization of morphologic features of apoptotic cell death, we performed 4′,6-diamidino-2-phenylindole (DAPI) staining following inoculation of NCI-H929 cells with 1 and 2 × IC_50_ of TCF and ACF for 48 h. Figure 2 showed clear apoptotic features such as apoptotic bodies formation, chromatin condensation, nuclear fragmentation, as well as cell shrinkage.

**Figure 3:**
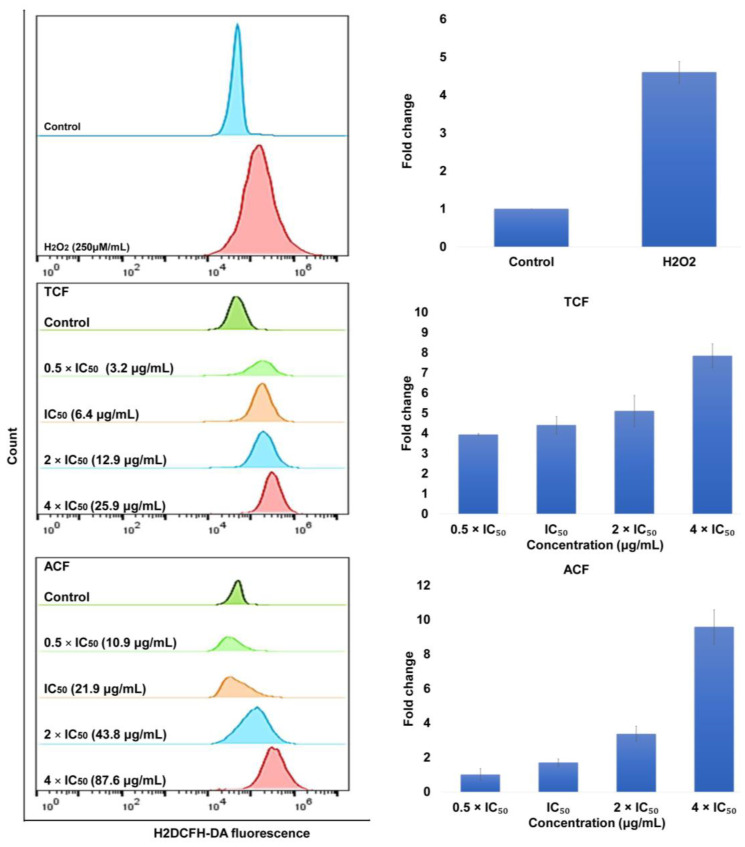
Intracellular production of reactive oxygen species in NCI-H929 cells. Results following 1 h incubation with 0.5, 1, 2, 4 × IC_50_ of chloroform fractions of *T. vulgaris* (TCF) and *A. lappa* (ACF), stained with 2′,7′-dichlorodihydrofluorescein diacetate (H2DCFH-DA) and analyzed by flow cytometry. Control: NCI-H929 cell without treatment.

To find out whether TCF- and ACF-induced apoptosis was correlated with reactive oxygen species (ROS) generation and breakdown of mitochondrial membrane potential (MMP), we assessed ROS level and MMP integrity. After exposure of NCI-H929 cells to 0.5, 1, 2, 4 × IC_50_ concentrations of TCF and ACF stained with 2′,7′-dichlorodihydrofluorescein diacetate (H2DCFH-DA), we observed obvious elevations of ROS levels in treated cells compared to untreated cells. As presented in Figure 3, ROS generation was elevated by 7.8-, 9.5-, and 4.6-fold following 1 h treatment with TCF, ACF, or hydrogen peroxide H_2_O_2_ (as positive control), respectively.

**Figure 4:**
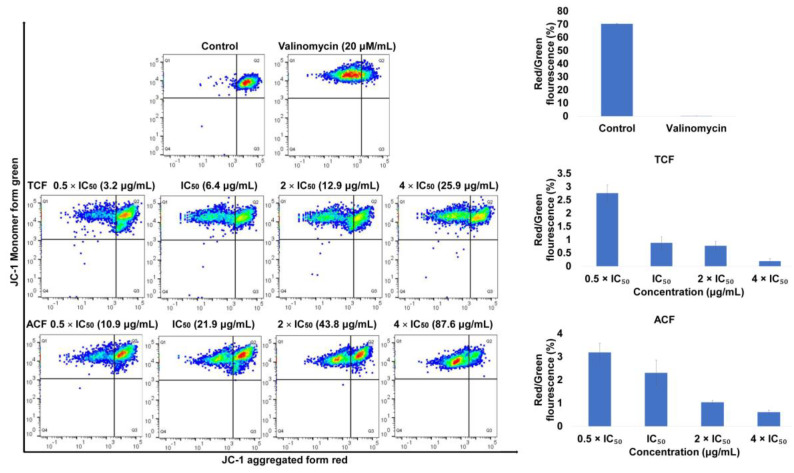
Disruption of mitochondrial membrane potential (MMP) in NCI-H929 cells exposed to 0.5, 1, 2, 4 × IC_50_ of chloroform fractions of *T. vulgaris* (TCF) and *A. lappa* (ACF) for 24 h, stained with 5,5′,6,6′-tetrachloro-1,1′,3,3′-tetraethylbenzimidazolyl carbocyanine iodide (JC-1) and analyzed by flow cytometry. The ratio of red (JC-1 aggregate)/green (JC-1 monomer) fluorescence was used to display the effect of TCF, ACF, and valinomycin on the MMP integrity. Control: NCI-H929 cell without treatment.

The loss of MMP is indicated by a decrease in the ratio of red/green fluorescence intensity. Figure 4 shows the addition of TCF and ACF at 0.5, 1, 2, 4 × IC_50,_ as well as valinomycin (positive control), for 24 h reduced the ratio of red (JC-1 aggregates) fluorescence to green (JC-1 monomers) fluorescence in a dose-dependent manner and the minimum ratio revealed on exposure to TCF (4 × IC_50_: 0.19%), followed by valinomycin (20 μM: 0.2%) and ACF (4 × IC_50_: 0.61%) in comparison to untreated NCI-H929 cells (70.42%). These findings indicate that apoptosis promoted by *T. vulgaris* and *A. lappa* was associated with ROS generation and disruption of MMP integrity.

### 2.3. Effect of TCF and ACF on Cell Cycle Distribution

**Figure 5:**
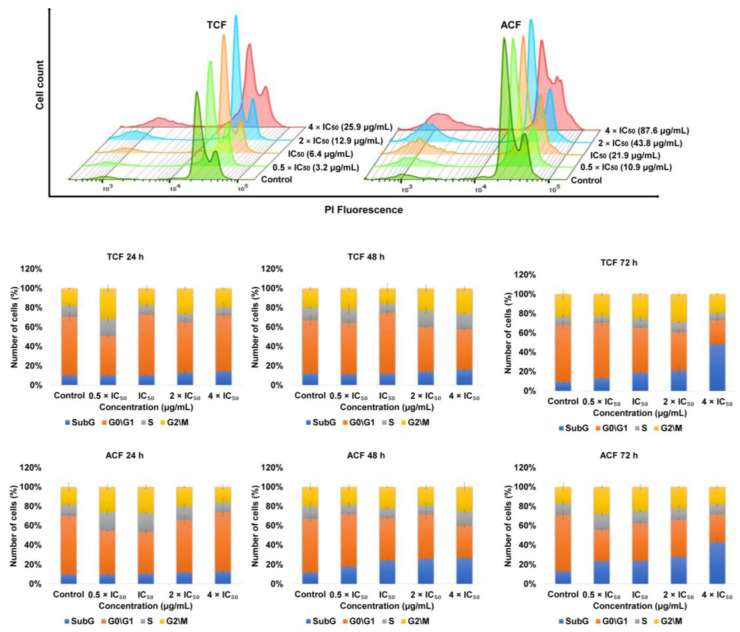
Induction of cell cycle disturbance in NCI-H929 cell lines treated with 0.5, 1, 2, 4 × IC_50_ of chloroform fractions of *T. vulgaris* (TCF) and *A. lappa* (ACF) following 24, 48, and 72 h, stained with propidium iodide (PI) and analyzed by flowcytometry. Control: NCI-H929 cell without treatment.

NCI-H929 cells were treated with various concentrations of TCF and ACF for 24, 48, and 72 h, stained with propidium iodide (PI) and analyzed using flow cytometry. The results in Figure 5 for TCF show that the cells accumulated in the (apoptotic) sub-G0/G1-phase (14.4%, 16% and 48.4%, vs. 10%, 12%, and 9.25% for untreated cells) following 24, 48, and 72 h, respectively. Furthermore, the cells arrested in the G2/M-phase (20% and 26%, vs. 18% and 20% for untreated cells) after 24 and 48 h, respectively, with corresponding decreases in proportion of cells arrested in the G0/G1-phase (57.5%, 42%, and 24.4% vs. 60.7%, 55%, and 59% for untreated cells). Additionally, the exposure of NCI-H929 cells to ACF resulted in arresting of cells in the sub-G0/G1-phase (13%, 26%, and 42.8%, vs. 10%, 12%, and 13% for untreated cells) following incubation for 24, 48, and 72 h, respectively, and G2/M-phase (25.1% and 19%, vs. 20% and 17% for untreated cells) after 48 and 72 h, respectively, with corresponding decreases in the ratio of cells in the G0/G1-phase (59%, 33%, and 28.7% vs. 60.7%, 55%, and 58% for untreated cells).

### 2.4. TCF and ACF Affects the Expression of Beclin-1 and LC3B-II

**Figure 6:**
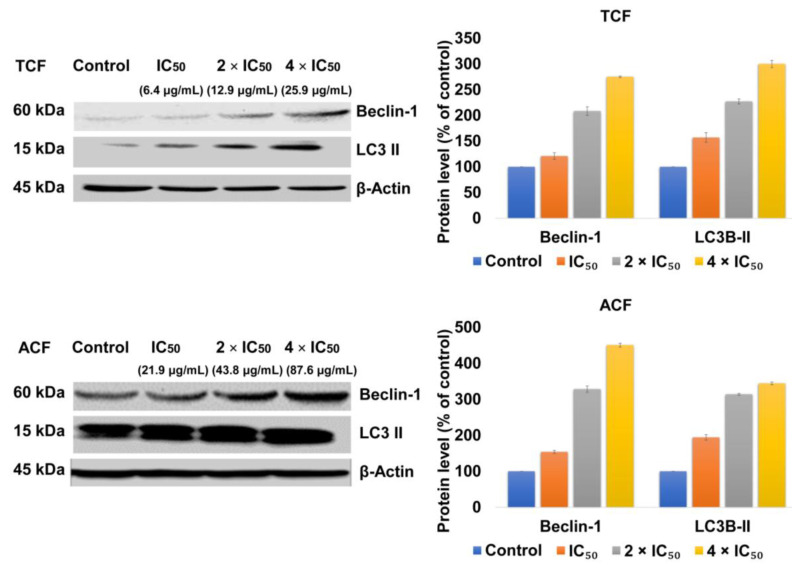
The expression of Beclin-1 and LC3B-II in human NCI-H929 cells treated with 1, 2, 4 × IC_50_ of chloroform fractions of *T. vulgaris* (TCF) and *A. lappa* (ACF) for 24 h was detected by western blot analyses. The relative protein expression of Beclin-1 and LC3B-II was normalized to β-actin. Control: NCI-H929 cell without treatment.

Following the treatment of NCI-H929 cells with TCF or ACF (1, 2, or 4 × IC_50_) for 24 h, the results in Figure 6 showed an elevated expression of Beclin-1 and LC3B-II compared to untreated control.

### 2.5. Phytochemical Study of TCF and ACF

**Figure 7:**
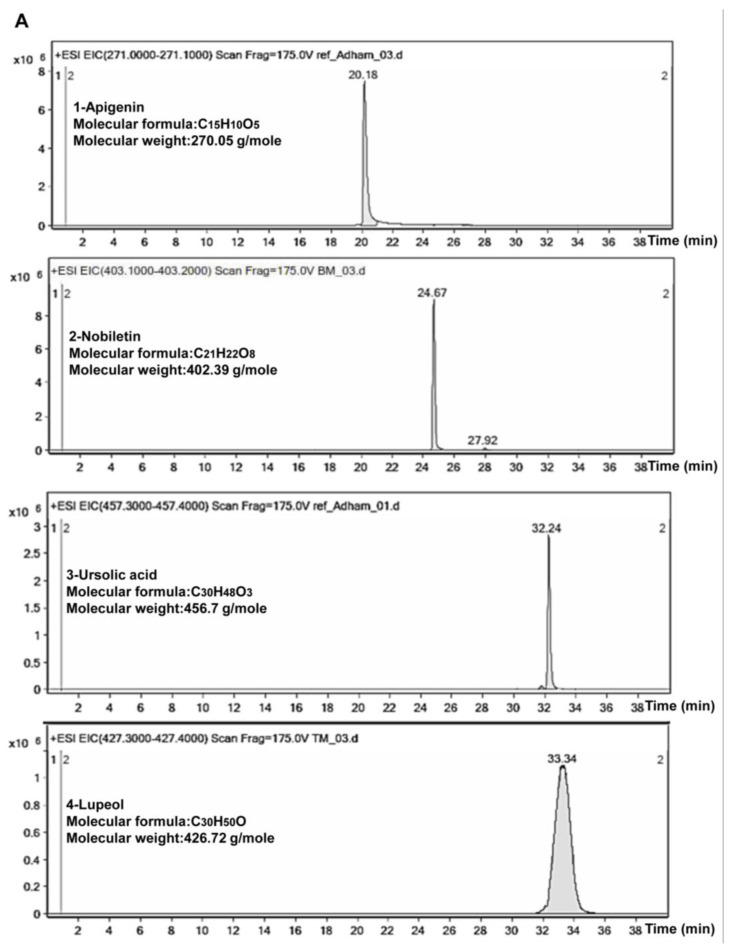

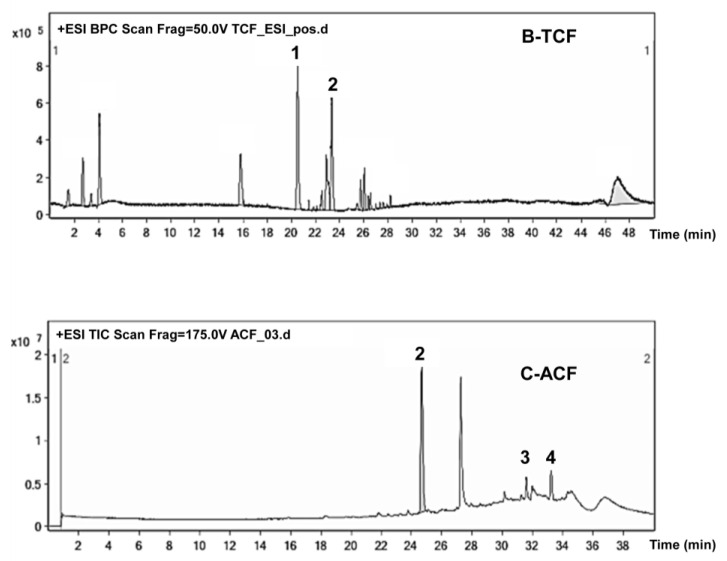
LC-ESI/MS chromatograms *T. vulgaris* and *A. lappa* extracts. (**A**)-Standard compounds. (1) Apigenin, (2) Nobiletin, (3) Ursolic acid, and (4) Lupeol. (**B**)-*T. vulgaris* chloroform fraction (TCF) and (**C**)-*A. lappa* chloroform fraction (ACF).

Liquid chromatography-electrospray ionization-mass spectrometer (LC-ESI/MS) analysis for TCF and ACF demonstrated the presence of 5–20 constituents in chloroform fractions of each plant. In Figure 7, the chromatogram of TCF revealed that the peaks no. 1 and 2 were predicted to be apigenin and nobiletin, while in the chromatogram of ACF peaks no. 2, 3, and 4 were predictive to be nobiletin, ursolic acid, and lupeol. These compounds were identified by comparison of our *m/z* values, MS/MS spectra, and the chemical formula by concomitant injection with authentic standard compounds and compared their retention time values.

## 3. Discussion

The cytotoxic activity of *T. vulgaris* and *A. lappa* towards various cancer cell lines was previously reported [11,12,19]. Several reports provided evidence on the activity against cell lines derived from solid tumors but very few data were available about their activity on MM cell lines. In our study, four fractions of *T. vulgaris* and *A. lappa* with different polarities were screened using the resazurin reduction assay against drug-sensitive CCRF-CEM and multidrug-resistant P-glycoprotein-overexpressing CEM/ADR5000 cells. All fractions showed cytotoxic activity against both cell lines with IC_50_ value between 2.13 ± 3.77 to 94.35 ± 4.60 μM/mL. Doxorubicin exhibited cytotoxic activity against sensitive and resistant phenotypes of leukemia cells such as CCRF-CEM cells and CEM/ADR5000 with IC_50_: 0.02 ± 0.00 and 122.96 ± 10.94 μM/mL [22]. CEM/ADR5000 were more sensitive to *T. vulgaris* and *A. lappa* extracts than Doxorubicin. According to the American National Cancer Institute, plant extracts with IC_50_ values < 30 μg/mL following 72 h incubation can be considered as reasonable strong cytotoxic activity [23]. Chloroform and ethyl acetate fractions of both plants met the required criteria for IC_50_ values in the range between 2.13 and 29.80 μg/mL for CCRF-CEM and CEM/ADR5000 cells. Thus, leukemia cell lines were highly sensitive to semi-polar compounds present in chloroform and ethyl acetate fractions as compared to polar and nonpolar compounds [24]. In another report, extracts of *T. vulgaris* revealed a dose-dependent reduction of THP-1 leukemia cell viability with an IC_50_ value of 156.9 μg/mL, while the toxicity towards normal human PBMCs was much less (IC_50_: 334.5 μg/mL) [11]. In our analyses, TCF and TEF revealed the highest cytotoxicity towards NCI-H929 cells with IC_50_ values of 6.49 ± 1.48 μg/mL and 25.55 ± 3.78 μg/mL, respectively. Doxorubicin prevents human MM cell lines such as RPMI 8226, U266, and NCI-H929 via apoptosis induction [25]. Cell death typically occurs by apoptosis, necrosis, ferroptosis, autophagy, pyroptosis, mitotic catastrophe, and so on. Apoptosis, also known as programmed cell death, a characteristic form of cell death, is controlled, energy-dependent and no inflammation is accompanying it [26]. TCF suppressed cell growth, induced apoptosis (late apoptosis) mainly accompanied by disruption of MMP integrity, affected cell cycle phase especially sub-G0/G1 in dose-time dependent manner in NCI-H929 cell lines, and slightly affected the G2/M phase at various concentrations. These results are in line with previous reports suggesting that polyphenolic extracts from *T. vulgaris* inhibited cell viability and promoted apoptosis in neuroblastoma cells, e.g., SH-SY5Y and SK-N-BE(2)-C cell lines at doses of 62.5 and 125 µg/mL [27]. Furthermore, the ethanolic extract arrested the cell cycle at the G2/M phase of T-47D breast cancer cells [28]. On the other hand, ACF displayed the greatest cytotoxicity against RPMI-8226 cells (IC_50_: 18.26 ± 0.26 μg/mL) and AEF against NCI-H929 cells (IC_50_: 35.01 ± 0.94 μg/mL). ACF inhibited cell growth, promoted necrosis and apoptosis (late apoptosis) that was mainly associated with ROS generation and induction of cell cycle arrest at a sub-G0/G1 phase in NCI-H929 cell lines, and at the same time slightly affected G2/M phase. However, previous studies reported that an ethanol extract of *A. lappa* root exhibited profound cytotoxic potency against Jurkat T-cell leukemia cells upon treatment for 24 h (IC_50_: 102.2 ± 42.4 μg/mL), by DNA fragmentation and induction of intrinsic apoptosis associated with loss of MMP and activation of caspase-3/7 without toxicity towards non-cancerous murine embryonic 3T3 fibroblasts [29]. *A. lappa* induced G0/G1 cell cycle arrest in gastric cancer cell lines as well as disturbance of the G2/M phase in colon cancer cells [30]. Our study showed that ferroptosis cell death in addition to apoptosis was involved in the tumor-suppressive activities of TCF and ACF. To the best of our knowledge, this is the first report regarding ferroptosis for these two plants. Ferroptosis is a novel form of cell death that is programmed necrosis and for the first time proposed in 2012 by Dixon [31,32]. It plays an essential role in the cessation of tumorigenesis by eliminating the cells that are scratched by infection or lacking in the main nutrients in the environment. Several studies have shown that the chief causative factor in triggering ferroptosis is the classic oxidative stress pathway. Erastin was the first introduced ferroptosis-inducing agent, followed by cisplatin, temozolomide, artesunate, sulfasalazine [33]. Interestingly, culturing NCI-H929 cells treated with TCF or ACF showed an upregulation of important autophagy-related markers such as LC3B-II and Beclin-1, implying the ability of both plants to induce autophagy. Beclin1 the mammalian orthologue of yeast Atg6, contributes as a scaffold, in the initial step for the autophagy process, which is the construction of phosphatidylinositol 3 kinases (PI3K) complex and present on the human chromosome 17q21. Beclin1 overexpression suppressed proliferation, and reduced the cell viability, by triggering autophagic cell death via the evolutionarily conserved domain and the coiled-coil domain bind to Vps34p and UVRAG, respectively [34]. LC3B-II belongs to the microtubule-associated protein 1A/1B-light chain 3 (LC3) family and accumulates particularly on nascent autophagosomes. Cytosolic-associated protein light chain 3 (LC3-I) converted to the membrane-bound LC3-II form on autophagosome formation [35]. ACF possessed higher efficacy compared to TCF. Apigenin and nobiletin were identified in the TCF, while nobiletin, lupeol, and ursolic acid were detected in ACF. The available literature demonstrated that flavonoids and terpenoids are abundant in vegetables, fruits, and medicinal herbs and revealed cytotoxicity towards several human cancer cell lines and induced cell death through various mechanisms [36]. A recent study reported that in MM cells, apigenin treatment potently inhibited cell development in a concentration-dependent manner by inducing cell apoptosis, ferroptosis, and autophagy, associated with down-regulating of STAT1, and Akt with concomitant activation of caspases, JNK, P-38 MAPK, Beclin-1, and LC3 II [37]. In human papillary thyroid carcinoma BCPAP cells, apigenin induced G2/M cell cycle arrest and DNA damage through suppressing the expression of Cdc25c and triggering the accumulation of ROS production [38]. Nobiletin repressed the development of human gastric TMK-1 cell lines, (IC_50_: 134.8 μM) through induction of apoptosis and cell cycle arrest in the G0/G1 phase [39], and suppress the expression of the crucial factor for endoplasmic reticulum stress such as thioredoxin-interacting protein (TXNIP), and consequently leads to cell apoptosis in human neuroblastoma cells [40]. Ursolic acid promoted apoptotic cell death in human breast cancer MCF-7 cells by Bcl-2 downregulation and suppressing the expression of transcription factor FoxM1 [41], while in MDA-MB-231 cells via mitochondrial death and extrinsic death receptor pathway [42]. Another study stated that ursolic acid triggered autophagy cell death in glioma U87MG cells through the formation of acidic vesicular organelles, the development of autophagolysosomes, and the accumulation of LC3-II [43]. Lupeol has shown cytotoxic activity toward MM RPMI 8226 (IC_50_: 50 μM), lung carcinoma A-549 (IC_50_: 50 μM), breast carcinoma MCF-7 (IC_50_: 50 μM), malignant melanoma G361 (IC_50_: 50 μM), and cervical carcinoma HeLa (IC_50_: 37 μM), following 72 h incubation [44]. Apoptosis induction of lupeol in human promyelotic leukemia HL-60 cells exhibited through the creation of hypodiploid nuclei and fragmentation of DNA in a concentration and time-dependent way [45].

## 4. Material and Methods

### 4.1. Chemicals and Reagents

Solvents such as *n*-hexane, chloroform, ethyl acetate, butanol, acetonitrile, and methanol were purchased from Chem-Lab (Zedelgem, Belgium). Penicillin and streptomycin were obtained from Gibco (Co Dublin, Ireland). Annexin V-FITC/PI detection apoptosis kit was obtained from Life Technologies (Carlsbad, CA, USA) and 5,5′,6,6′-tetrachloro-1,1′,3,3′-tetraethylbenzimidazolyl carbocyanine iodide (JC-1) kits from Biomol (Hamburg, Germany). H2DCFH-DA, H_2_O_2_, ferrostatin-1, deferoxamine, valinomycin, PI, resazurin, triton X-100, paraformaldehyde, tween-20, dimethylsulphoxide (DMSO), DAPI and doxorubicin (98.0% purity) were purchased from Sigma-Aldrich (Taufkirchen, Germany). M-PER^®^ mammalian protein extraction reagent and protease inhibitor were obtained from Thermo Fisher Scientific (Waltham, MA, USA). Antibodies against Beclin-1 (D40C5), LC3B, β-actin (D6A8) and anti-rabbit IgG HRP-linked antibody were purchased from Cell Signaling Technology (Danvers, MA, USA) and Luminata™ Classico Western HP substrate was obtained from Merck Millipore (Darmstadt, Germany).

### 4.2. Preparation of Plant Extracts

Aerial parts of *T. vulgaris* and roots of *A. lappa* were collected from the Kurdistan region, Iraq, during 2018–2019. The plant materials were authenticated by assistant professor Al-Khayat AH, and vouchers (A-11 and A-12, respectively) were deposited at Pharmacognosy Department, Pharmacy College, Hawler Medical University. Powdered plant materials (each 500 g) were extracted successively in a Soxhlet extractor [46] with methanol for 36 h. The extracts were concentrated to dryness by using a rotary vapor machine at 40–50 °C (Buchi Rotavator^®^, Switzerland) and re-dissolved in 10% methanol, followed by liquid-liquid fractionation using (10×) with an equal volume of *n*-hexane, chloroform, ethyl acetate, and butanol solvents. The obtained fractions of HF, CF, EF, and BF were concentrated under vacuum and subsequently powdered using a freeze-dryer (Martin Christ Alpha 1–2 LD plus, Osterode am Harz, Germany) to provide fractions with diverse polarity. All fractions were kept at 4 °C for further investigations.

### 4.3. Cell Culture Conditions

Nine multiple myeloma cell lines, MOLP-8, NCI-H929, RPMI-8226, KMS-12BM, KMS-11, L-363, JJN-3, AMO-I, and OPM-2 were kindly provided by Ellen Leich-Zbat (Institute of Pathology, University of Würzburg, Germany) [47]. Sensitive CCRF-CEM and multidrug-resistant CEM/ADR5000 leukemia cells were obtained from Prof. Axel Sauerbrey (Department of Pediatrics, University of Jena, Germany). The multidrug resistance phenotype of CEM/ADR5000 cells has been previously characterized [21,48]. The cell lines were cultured in Roswell Park Memorial Institute (RPMI 1640) medium (Gibco, Co Dublin, Ireland), supplemented with 10% fetal bovine serum (FBS) (Gibco, Co Dublin, Ireland), and 1% penicillin (100 U/mL)-streptomycin (100 µg/mL) in a humidified atmosphere with 5% CO_2_ at 37 °C. The resistance of CEM/ADR5000 cell lines has been maintained by adding 5000 ng/mL doxorubicin. PBMCs were isolated and cultivated as reported previously [49].

### 4.4. Resazurin Reduction Assay

The cell viability of cells was studied by means of the cell-permeable redox indicator resazurin. Briefly, 1 × 10^4^ cells/well of MM and leukemia cells were suspended in 100 μL of the growth medium, plated in 96 well plates; then the volume was increased to 200 μL/well by treating the cells with 100 μL of medium containing various concentrations of plant extract (0.001–100 μg/mL), previously dissolved in DMSO (final concentration 0.3%). Cells treated with 0.3% DMSO were considered as a negative control [50]. The resazurin assay was also used to investigate the influence of ferroptosis inhibitors such as ferrostatin-1 and iron chelators deferoxamine on the cytotoxicity of various plant fractions as previously described [51]. Further, 1 × 10^4^ NCI-H929 cells/well were cultured with ferrostatin-1 (50 μM) and deferoxamine (0.2 μM) for 1 h before exposure to treatment. Subsequently, cells incubated with different concentrations of plant fractions (0.001–100 μg/mL) and control. After 72 h incubation in a 5% CO_2_ environment at 37 °C, 20 µL 0.01% *w/v* resazurin solution was added to each well and incubated in the dark for a further 4 h. The resazurin fluorescence was measured at an excitation wavelength 544 nm and emission at 590 nm using an Infinite M2000 Pro^TM^ plate reader (Tecan, Crailsheim, Germany). The IC_50_ of different cell lines was calculated from a calibration curve by exponential linear regression using GraphPad Prism6 software and expressed in mean ± SD. The degree of resistance was determined as the IC_50_ value of the resistant cell line over the IC_50_ value of the sensitive cell line [22]. The experiments were repeated three times.

### 4.5. Flow Cytometric Assessment of Apoptosis

NCI-H929 cells (10^6^ cells/well) were treated for 48 or 72 h with various concentrations of TCF and ACF, then incubated at 37 °C and 5% CO_2_ atmosphere. Cells were harvested and resuspended in Annexin V-binding buffer. Subsequently, 5 µL Annexin V-FITC and 10 µL PI were added and incubated for 15–20 min in the dark place and room temperature [52]. Cell apoptosis and necrosis were determined using BD Accuri™ C6 flow cytometer (BD Biosciences, Heidelberg, Germany). The results evaluated using FlowJo software version 7.0. and each experiment was conducted three times. The data expressed as the percentage of cells in each population (viable cell annexin V−/PI−; early apoptotic annexin V+/PI−; late apoptotic annexin and early necrotic V+/PI+; late necrotic V−/PI+).

### 4.6. Flow Cytometric Assessment of Mitochondrial Membrane Potential

Briefly, 24 h after plating the NCI-H929 cells (10^6^ cells/well)with various concentrations of TCF and ACF, 0.3% DMSO (negative control) and valinomycin (positive control), at 37 °C and 5% CO_2_ incubation, cells were then incubated with JC-1 for 30 min. The cells were analyzed using the LSR-Fortessa FACS analyzer (Becton-Dickinson, Heidelbeg, Germany) and ratio of red/green fluorescence intensity was used to determine alteration in integrity of the MMP and calculated using FlowJo software version 7.0 [53,54]. Each observation was conducted three times.

### 4.7. Flow Cytometric Assessment Reactive Oxygen Species

NCI-H929 cells (2 × 10^6^ cells/well) were resuspended in phosphate buffer saline (PBS) (Gibco, Co Dublin, Ireland) and exposed to 2 μM of H2DCFH-DA for 30 min. Subsequently, the cells were treated with various concentrations of TCF and ACF, H_2_O_2_ (positive control), or DMSO (negative control). After incubation for 1 h, the treated cells were washed twice to remove the extracellular compound and suspended in PBS. The 2′,7′-dichlorofluorescein fluorescence was detected using flow cytometry [55]. The amount of ROS achieved was calculated using FlowJo software version 7.0. Each experiment was conducted three times.

### 4.8. Flow Cytometric Assessment of Cell Cycle Distribution

NCI-H929 cells (10^6^ cells/well) were incubated with various concentrations of TCF and ACF for 24, 48, and 72 h at 37 °C and 5% CO_2_. DMSO (0.3%) was used as a negative control. Cells were harvested and fixed by adding 1 mL cold absolute ethanol gradually with vortexing to the cell pellet and stored at −20 °C for 24 h. The cells were harvested and washed with PBS, and the nuclei were stained with PI at a final concentration of 50 μg/mL. The percentage of DNA contents in each phase (sub-G0/G1, G0/G1, S and G2/M) was measured using a BD Accuri™ C6 flow cytometer [56]. Each experiment was conducted three times.

### 4.9. Fluorescence Microscopy

Following 48 h treatment of NCI-H929 cells (10^6^ cells/well) with various concentrations of TCF and ACF, the cells were fixed with 4% paraformaldehyde for 30 min at room temperature, washed with PBS, and blocked with 5% FBS and 0.1% Triton X-100 in PBS for a further 1 h at room temperature. The nuclear morphology of apoptotic cells was monitored by staining cell nuclei with 1 µg/mL of DAPI [57], in the dark for 10 min, at 37 °C and visualized under a fluorescent microscope (EVOSs FL Cell Image System, Thermo Fisher Scientific).

### 4.10. Western Blotting

NCI-H929 cells (10^6^ cells/well) were treated with various concentrations of TCF and ACF or 0.3% DMSO as a negative control. Following 24 h incubation, the cells were lysed with Mammalian Protein Extraction Reagent M-PER^®^ and protease inhibitor at ratio (1:100) at 4 °C for 30 min. Protein concentrations were determined by Nano-Drop1000 spectrophotometer (Thermo Fisher Scientific). Isolated protein was mixed with sodium dodecyl sulfate-polyacrylamide gel electrophoresis (SDS-PAGE) loading dye and boiled at 95 °C for 10 min. The equal quantity of protein (30 µg) was subjected to 10% SDS-PAGE and subsequently transported to polyvinylidene difluoride membranes (RotiR PVDF, pore size 0.45 µm, Carl Roth GmbH, Karlsruhe, Germany). Further, 5% (*w*/*v*) bovine serum albumin (Sigma-Aldrich, Darmstadt, Germany) in Tris-buffered saline containing 0.5% tween-20 (BSA/TBST) was used to block the non-specific binding sites in the membranes at room temperature for 1 h. The membranes were incubated with antibodies against LC3B, Beclin-1 or β-actin (as normalizing control) overnight at 4 °C. After washing with TBST, the membranes were further incubated with HRP-linked secondary anti-rabbit antibody for 1 h at room temperature. The detection of protein band was carried out by exposing the membrane to Luminata™ Classico Western HRP substrate, and images of the membranes were captured with the Alpha Innotech Fluor Chem Q system (Biozym, Germany) [58]. Image Studio Lite Software was used for the determination of relative band intensity.

### 4.11. LC-ESI/MS Analysis of T. vulgaris and A. lappa Fractions

TCF and ACF were analyzed by LC-ESI/MS system. The high-performance liquid chromatography (HPLC) system was 1260 Infinity II (Agilent Technologies, Waldbronn, Germany). A reversed-phase Eclipse Plus C_18_ RRHP (50 × 2.1 mm, 1.8 μm particle size, from Agilent Technologies) was used for the separation process. The eluents consisted of 2% acetonitrile in H_2_O (Phase A) and 100% methanol (Phase B), and the following gradient mode was used: 0–1 min, 15% B isocratic; 1–24 min, linear gradient from 15% to 95% B; 24–29 min, 95% B isocratic; 29–30 min, linear gradient from 95% to 15% B; and re-equilibration 30–50 min, 15% B isocratic. The column temperature was set to 30 °C, the flow rate to 0.2 mL/min, and the injection volume of 5 µL. HPLC coupled to a 6545 quadrupole time of flight mass spectrometer (Agilent Technologies) equipped with an Agilent Jet Stream electrospray ionization interface working in the positive mode. High-purity nitrogen was used as the nebulizer and auxiliary gas, and conditions were set at drying gas temperature 320 °C, sheath gas temperature 350 °C, and a flow rate of 10 L/min. The nebulizer pressure set to 35 PSIG, capillary voltage 3.5 kV, nozzle voltage 1 kV. The fragment was set to 175 (arbitrary units). Full MS scans from *m/z* 100–3000 Da were acquired at a scan rate of 1 spectrum/s. Data processing was performed using mestrenova software.

## 5. Conclusions

Different fractions of *T. vulgaris* and *A. lappa* inhibited proliferation and reduced cell viability of leukemia and MM cell lines rather than normal human PBMCs in a concentration-dependent manner. As a consequence, our study demonstrated that both plants possess potent cytotoxic activity against hematologic malignancies, the results were supported by an evaluation of their mechanism of action. Among various fractions, TCF and ACF as potent cytotoxic fractions induced apoptosis (as shown in the sub-G0/G1 fraction) and slightly arrested cell cycle progression in the G2/M phase. TCF triggered late apoptosis and early necrosis, while ACF triggers late apoptosis and both early and late necrosis mediated by MMP disruption and increased intracellular ROS levels, which was further emphasized by distinctive morphological changes. The autophagic effects of ACF were greater than that of TCF. In addition to the mentioned mechanisms, ferroptosis cell death was also promoted by TCF and ACF in NCI-H929 cells. LC-ESI/MS analysis exhibited a variety of chemical constituents in TCF and ACF, four of which were identified as apigenin, nobiletin, lupeol, and ursolic acids.

## Figures and Tables

**Table 1:** The proportion of different solvent extractions of *T. vulgaris* and *A. lappa.*

Plants	Solvents	Yields (*w*/*w* %)	Color	Consistency
***T. vulgaris***	*n*-Hexane	21.172	Dark green	Greasy, semisolid
	Chloroform	2.663	Dark green	Solid
	Ethyl acetate	28.236	Pale yellow	Solid
	Butanol	41.507	Dark red	Gummy
***A. lappa***	*n*-Hexane	1.828	Yellowish green	Greasy, semisolid
	Chloroform	0.454	Reddish brown	Solid
	Ethyl acetate	4.217	Reddish brown	Solid
	Butanol	12.756	Dark red	Gummy

**Table 2:** Cytotoxicity of different *T. vulgaris* and *A. lappa* fractions towards leukemia cell lines as determined by resazurin assay.

	*T. vulgaris*			*A. lappa*		
Fractions	CCRF-CEM	CEM/ADR5000		CCRF-CEM	CEM/ADR5000	
	IC_50_ (μg/mL ± SD)	IC_50_ (μg/mL ± SD)	D.R.	IC_50_ (μg/mL ± SD)	IC_50_ (μg/mL ± SD)	D.R.
**HF**	31.51 ± 1.63	34.02 ± 0.84	1.08	29.72 ± 1.10	55.93 ± 0.68	1.88
**CF**	2.13 ± 3.77	4.00 ± 0.15	1.88	6.75 ± 0.95	14.95 ± 3.28	2.21
**EF**	4.35 ± 1.18	24.85 ± 2.60	5.71	25.38 ± 3.29	29.80 ± 2.32	1.17
**BF**	28.64 ± 0.35	94.35 ± 4.60	3.29	30.67 ± 2.09	93.48 ± 4.89	3.05

D.R., degree of resistance; HF, *n*-hexane fraction; CF, chloroform fraction; EF, ethyl acetate fraction; BF, butanol fraction. Values are presented as the mean ± SD.

**Table 3:** Cytotoxicity of chloroform and ethyl acetate fractions of *T. vulgaris* and *A. lappa* towards MM cell lines as determined by the resazurin assay.

MM Cell Line	*T. vulgaris*		*A. lappa*	
	IC_50_ (µg/mL ± SD)		IC_50_ (µg/mL ± SD)	
	CF	EF	CF	EF
**MOLP-8**	13.45 ± 3.49	41.63 ± 0.53	39.51 ± 2.30	56.92 ± 1.00
**NCI-H929**	6.49 ± 1.48	25.55 ±3.78	21.9 ± 0.69	35.01 ± 0.94
**RPMI-8226**	27.14 ± 0.01	30.17 ± 0.17	18.26 ± 0.26	>100
**KMS-12BM**	15.28 ± 4.90	31.78 ± 3.46	22.3 ± 0.18	81.2 ± 1.78
**KMS-11**	17.26 ± 2.48	35.15 ± 1.93	31.95 ± 2.37	65.04 ± 1.89
**L-363**	11.28 ± 4.64	25.61 ± 2.21	46.97 ± 3.66	35.62 ± 0.43
**JJN-3**	13.88 ± 1.19	30.11 ± 1.69	25.99 ± 0.70	48.00 ± 4.41
**AMO-I**	14.02 ± 2.64	35.07 ± 3.23	29.11 ± 1.04	35.02 ± 0.59
**OPM-2**	6.91 ± 3.70	26.06 ± 0.78	35.63 ± 4.079	45.96 ± 2.49

MM, multiple myeloma; CF, chloroform fraction; EF, ethyl acetate fraction. Values are presented as the mean ± SD.
